# Looking for the “Little Things”: A Multi-Disciplinary Approach to Medicines Monitoring for Older People Using the ADRe Resource

**DOI:** 10.3390/geriatrics5040079

**Published:** 2020-10-19

**Authors:** David Hughes, Meirion Jordan, Patricia A. Logan, Alan Willson, Sherrill Snelgrove, Melanie Storey, Mojtaba Vaismoradi, Sue Jordan

**Affiliations:** 1 Faculty of Health and Life Sciences, Swansea University, Swansea SA2 8PP, UK; d.hughes@swansea.ac.uk (D.H.); a.r.willson@swansea.ac.uk (A.W.); s.r.snelgrove@swansea.ac.uk (S.S.); m.storey@swansea.ac.uk (M.S.); 2 Independent Researcher, Swansea SA9 2GA, UK; meirion.jordan@googlemail.com; 3 Faculty of Science, Charles Sturt University, Bathurst 2795, Australia; plogan@csu.edu.au; 4 Faculty of Nursing and Health Sciences, Nord University, 8049 Bodø, Norway; mojtaba.vaismoradi@nord.no

**Keywords:** adverse drug reactions, patient safety, nursing, medicine management, long-term care, community care, older people care

## Abstract

Advances in medicines have increased the effectiveness of treatments and the social and cultural authority of doctors. However, as prescribing has become the dominant modality of treatment, the “pharmaceuticalization” of medical practice has often resulted in treatment “at a distance”, with doctors having limited contact with patients. Older and poorer people, who are socially distanced from medical prescribers, suffer more adverse drug reactions (ADRs) than the general population. A team approach to checking patients systematically for ADRs, as detailed in manufacturers’ literature, can minimise medication errors, but regular review is rare. This paper explains the benefits of medicines monitoring to protect older patients from iatrogenic harm, such as over-sedation, falls, or drug-induced Parkinsonism. We show how multidisciplinary initiatives to optimise prescribing can be supported by using a recognised resource—the adverse drug reaction profile (ADRe). The profile identifies and documents patients’ signs and symptoms of putative ADRs. Better monitoring allows professionals to adjust prescribing and respond to identified problems with agility. Implementation of systematic monitoring will require changes to the regulatory regime and better inter-professional cooperation. Providing carers, nurses and pharmacists with a structured system to monitor patients would democratise relevant medical knowledge and help address ageism and the socio-economic health divide.

## 1. Introduction

The benefits of improved medicines developed over the past century [1,2] have been eroded by an increasing incidence of iatrogenic disease arising with the unwanted effects of prescribed drugs [3,4]. The European Medicines Agency [5] defines an adverse drug reaction (ADR) as a “noxious and unintended” reaction to a medicinal product. This includes ADRs that arise from both use of a medicinal product within the terms of the marketing authorisation, and use outside the terms of the marketing authorisation, including overdose, off-label use, misuse, abuse and medication errors, as well as occupational exposure [5] (page 6).

ADRs are amongst the most common forms of iatrogenic harms, disproportionately affecting the elderly and the poor and consequently presenting a serious challenge for contemporary healthcare systems. This challenge persists despite numerous regulatory and policy initiatives to improve quality of care, due in part to systemic power imbalances between prescribers, patients and non-prescribing healthcare professionals.

Our intervention, the adverse drug reaction profile (ADRe), allows care home staff periodically to record the signs and symptoms of adverse effects of commonly prescribed medicines by completing a paper instrument—a kind of carer administered checklist. It is structured so as to allow detection of common ADRs and provides nurses and other non-prescribers with information to link signs and symptoms on the checklist to prescription medicines. This offers personalised information and evidence that they can bring to the attention of prescribers so as to facilitate improved medicines management [6,7]. We named our profile after the Welsh language word “adre” means “homeward”, and implying a return to a place of safety where one can enjoy health and wellbeing. ADRe provides a systematic means of improving drug safety in primary care, while also encouraging new forms of multidisciplinary working that will help to reduce the “care gap” [8] that affects so many older care home residents.

## 2. Prescribing as an Expression of Professional Power

Over- and under-prescribing, and the ADR problem, need to be seen in the wider context of long-term changes in the practice of medicine. Recent decades have seen the increasing “pharmaceuticalization” of medical practice [9,10,11], whereby the dominant modality of treatment is the prescribing of often powerful medicines. Williams and colleagues (p. 711, [10]) define pharmaceuticalization as “the translation or transformation of human conditions, capabilities and capacities into opportunities for pharmaceutical intervention”. Over time, a growing range of human problems are seen as being amenable to medical treatment [12], which increasingly centres on pharmaceutical products, including medicines that control behaviour. Pharmaceuticalization typically leads to a form of “treatment at a distance”, a mode of physician engagement with patients where an initial contact leads to an extended course of treatment, the ongoing effects of which are either monitored by patients themselves or by other carers, and where further contact with the doctor is at best episodic. We have argued previously that despite modern efforts to move towards “patient-centred medicine”, medical professionals often remain socially distant from certain sections of the population, especially older patients in long-term care facilities [13]. The combination of social distance between doctors and older patients, and treatment centring on drugs prescribed without regular patient contact, increases the risks of medication errors and undetected ADRs. Past studies have found that medical care in nursing homes remains suboptimal; in particular, the inappropriate use of psychotropic medication and shortcomings in pain management remain key concerns in the care of older adults [14,15,16]. An effective medical monopoly on prescribing dis-incentivises others in the care team from undertaking the work of tracking adverse side effects. These ongoing risks are compounded by the “sick role” status and unintentional “othering” of patients [17], whereby patients risk being defined by their diagnoses and prescriptions and then confined to these powerless states by inadequate medicines management.

Given that the potential harms of prescribed medicines have been consistently underestimated, there is an urgent need for effective medicines management in the care of older adults [18,19]. One study found ADRs affected 7.8% of primary care patients [20], and in more vulnerable groups, this rises significantly. ADRs have been found to affect 4.8–37% of people with cognitive impairment [21] and 11.0% of hospitalised patients [22]. Drug-induced signs and symptoms are difficult to distinguish from underlying disease conditions, often leading to further prescriptions and increasing the risk of drug interactions [23,24]. Prescribing depends almost exclusively on the actions of medical professionals, but systematic monitoring to check for ADRs rarely occurs [25,26,27,28], and there is no generally-accepted tool for safeguarding patients from under- or over-prescribing [29].

## 3. ADRs in Older Adults: Systems Failure or Systemic Challenge

With prescribing taking place within the political context of a medical monopoly, it is concerning that ADRs remain a serious source of harm. Adverse effects of medical treatment (by no means all ADRs) were the underlying cause of an estimated 123,603 deaths in the USA between 1990 and 2016 [30]. Approximately 6% of patients are harmed by care processes, half of which are preventable [31]. Over recent decades, ADRs have been responsible for 5–8% of unplanned UK hospital admissions [32,33], rising to around 10% amongst older adults [34]. One English study [35] estimated that the cost of avoidable ADRs was at least £98.5 million per annum, and probably significantly higher, while another put the figure at between a billion and 2.5 billion pounds [36]. Moreover, the impacts of ADRs are often more prevalent among older adults, with all-cause mortality highest amongst those prescribed mental health medicines [4].

Both ADRs and failure to prescribe necessary medicines increase mortality and hospital admissions, and this underlines the central importance of prescribing for contemporary health care [37]. Population ageing and the increasing volume of drugs prescribed will worsen these problems. Doses for mental health medicines in care homes [38] and primary care [18] are often excessive, and research suggests that over half of care home residents may be affected [39]. ADRs from these sources can be life-threatening but are often ignored or mistaken for symptoms of ageing or underlying disease [6]. There remains strong evidence that most ADRs are preventable [25,37,38]. Additional enhanced monitoring is one component of the change needed to address this challenge [37,38,40,41,42,43,44]. We need comprehensive, systematic and multi-professional approaches to improve patient safety [29,45]. It is crucial to involve the wider care team rather than rely only on monitoring by prescribers.

## 4. ADRe and Systematic Medicines Management as a Route to Effective Change

The urgency of this problem is underlined by research that identifies medicine-related harms as the main source of unsafe primary care in England and Wales [46]. Nonetheless, the majority of ADRs and other medicine-related harms arise from poor monitoring rather than errors in prescribing [32,33,40,41,42,43], being primarily dose-related [44]. The situation can be greatly improved by enhanced monitoring and reporting [18,19,47]. ADRe was developed from earlier research on nurse-led medication checking, originally in relation to mental health medicines prescribed for clients of community mental health teams [48,49,50].

## 5. How Is ADRe Different?

**Figure 1:**
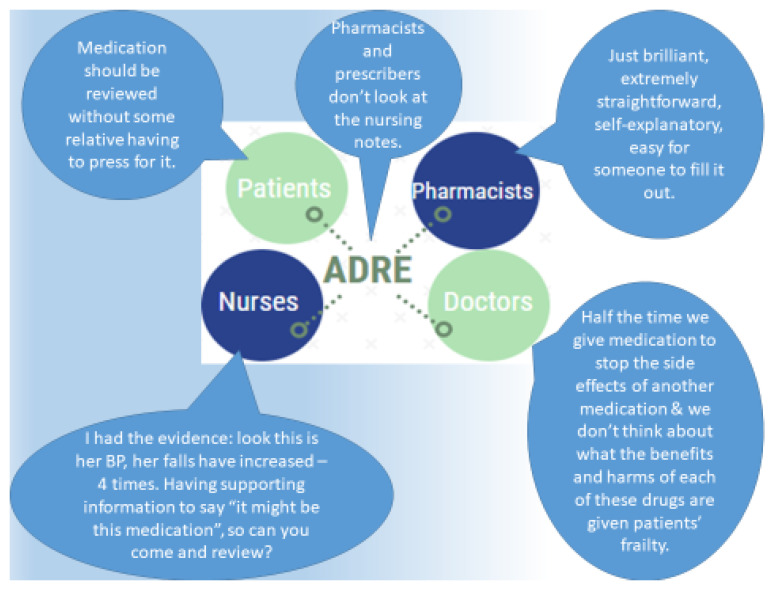
The adverse drug reaction profile (ADRe): some stakeholder opinions. These previously unpublished quotations come from 30 qualitative interviews with service users, care home staff and professionals completed in our 2019 study [6,7]. In brief, we interviewed nurses and residents (or family) from 10 care homes, as well as a range of stakeholders, including pharmacists and prescribing doctors, to identify the barriers and facilitators to using ADRe in real-world settings. The methods are detailed elsewhere [7].

ADRe represents a unique approach to assembling patient information, checking and optimising prescribing and preventing drug-related harm and hospital admissions. Staff use the paper instrument at intervals of approximately three months or before scheduled appointments with doctors or pharmacists to collect systematic information for each patient, including on signs and symptoms of known adverse effects of commonly prescribed medicines. The profile is designed to detect “undesirable“ side effects based on manufacturers’ summaries of product characteristics (SmPCs). The guide provided with ADRe suggests possible causes for each sign or symptom. Information collected is then shared with pharmacists and prescribers considering repeat prescriptions or reviewing medicines administration record (MAR) charts. ADRe thus represents a multidisciplinary intervention to complement wider regulatory initiatives seeking to improve medicines management and reduce errors. This is illustrated by stakeholders’ views (Figure 1). The authors’ review of the literature found no alternative comprehensive, systematic patient assessment tool for monitoring prescribed medicines [6,45].

While many interventions aid the selection of appropriate medication, few have demonstrated improvement in patient outcomes [32,34]. ADRe is different—it addresses the “little” things affecting patients, aiming to prevent serious adverse events, for example, by addressing dizziness, balance and poor eyesight before falls happen. The attention to detail that ADRe permits explains the improvement in symptoms such as pain, dyspnoea, sedation, aggression and confusion we found in our 2019 study [6].

## 6. What Does ADRe Entail?

ADRe focuses on systematic checks for an itemized set of adverse or undesirable effects of patients’ mental health medicines. It also asks care staff to take action in case of problems and, crucially, to share that information with pharmacists and prescribers. It formalises and standardises the approach to monitoring patients′ prescribed mental health medicines. Problems identified are then considered alongside the prescription record, and the multidisciplinary team reviews possible adverse effects and decides whether prescriptions need to be repeated, discontinued or changed. ADRe allows non-prescribing practitioners to present direct evidence for their concerns to prescribers. In our study of the implementation of ADRe in ten care homes [6,7], several respondents commented on the opportunity for greater engagement that the tool provided (see Figure 1):

“The CPN started a resident on lurasidone and after a week, we noticed her behaviour had escalated: she was restless, constipated, dehydrated, confused, disorientated, far worse than she had been, (…) I made some notes on it [ADRe], to lead me in the right directions, really to go down the right path. (…) Rather than just, trying to put my opinion across, I had the evidence in front of me, the things to say ‘actually, it might be this medication, so can you come and review?’ The CPN might have said ‘carry on’, but she said ‘ok, fair enough’, and came back and we had a good result. It’s useful, definitely useful, but quite complex to work on. She’s still quite challenging but we haven’t got all these issues, side effects, any more.” (Care home lead nurse, N6)

Pharmacists too commented on how the instrument had the potential to change patterns of communication between professionals and care home staff.

“Trying to get hold of the GPs in the first place... We have no direct line of communication with them. Some surgeries are extremely difficult to get hold of to get to the actual receptionist to get request a conversation with the GPs. That is a major barrier. When you can get hold of them, they [GPs] are absolutely fine. (…) A person could be absolutely fine when the nurse comes to visit and then for the rest of the day, they could be exhibiting some sort of symptom or side effect. So if the carers were able to use this [ADRe] it would be extremely valuable (…) and if they need help, to come to us rather than the GP.” (Retail pharmacist)

**Figure 2:**
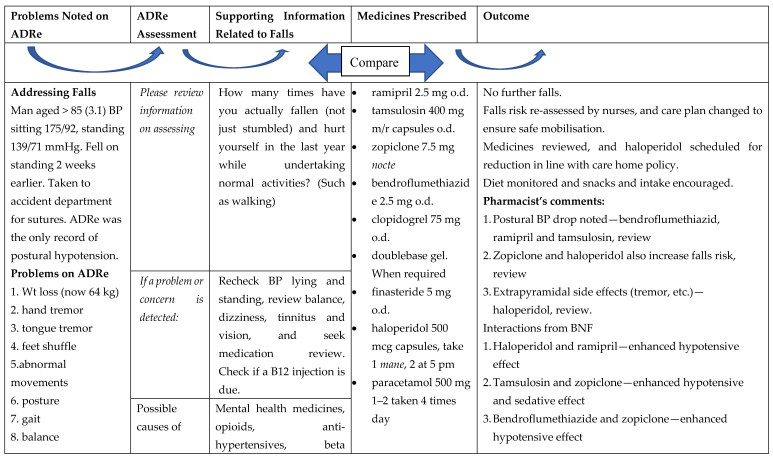

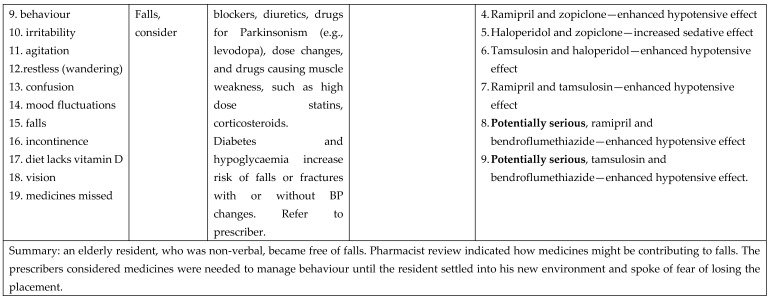
ADRe in action: preventing falls. ADRe: the adverse drug reaction profile, N.B. Medicines relevant to the resident are emboldened in the supporting information, BP = blood pressure, o.d. = omni die (every day).

ADRe thus functions to address the physical and social distance and difference in power between prescribers and other health care workers, improving communication and improving care. It also augments the role and increases the visibility of pharmacists, who, while having more training in therapeutics than any of the other professions, are often distanced from patients and their symptoms. Figure 2 uses a typical case to illustrate how ADRe identifies problems, which are then addressed. In this instance, prescribers felt that prescriptions should remain until the resident had settled into the care home; however, the risk of falls was addressed by nursing actions.

## 7. ADRe in the Context of Regulatory and Policy Interventions

ADRs are often unrecognised [13], but as we have seen they have very significant impacts in terms of increased morbidity, premature mortality and financial cost to the UK National Health Service (NHS). These impacts are sufficiently weighty and widespread that they require intervention at the policy level. It is clear that there is a pressing need for policy intervention to address the problem of ADRs in older adults [6,13].

Policy intervention is important to encourage care homes to get past the common initial perception that using a resource such as ADRe imposes a work burden that they cannot afford. Our study found that familiarity reduces the time taken to administer ADRe to around 10 min per patient each month or each quarter. Once familiarity is gained, routine use becomes relatively easy. The problem is that a push is needed to persuade care homes to take the first step. Our experience in Wales suggests that research evidence of health gain and even endorsement by authoritative bodies have, to date, been insufficient to ensure rollout beyond the most innovative care homes. ADRe has been endorsed by the Royal Pharmaceutical Society and recommended as best practice by the All Wales Medicines Strategy Group, but this has yet to impact on policy makers and practice. Disappointingly, the COVID-19 pandemic did not lead to uptake of a resource to monitor changes in oxygen saturation, temperature, cough (and respiratory symptoms) or changes in appetite and taste, where ADRe would prove very beneficial to longer term patients.

In our view, what might make a difference is for the statutory inspection bodies to encourage care homes to use a formal medicines monitoring tool. While Care Inspectorate Wales has a remit to monitor medicines administration in care homes, it takes the view that it has no power under existing government guidance to require homes to use particular monitoring tools. It does use an audit questionnaire, developed in cooperation with the chief pharmacist, to assess the general approach taken, but this is a blunt instrument for addressing the magnitude and complexity of the ADR problem. Including a question that asks whether homes utilise an evidence-based resource and regarding this as an indicator of good practice, would incentivize more homes to safeguard against iatrogenic harm. In the longer term, it may be possible to go a step further and change guidance to require use of a formal medicines monitoring tool in all registered care homes.

Changing practice will depend on mobilizing support for ADRe through persuasion within the key influence networks and grappling with the professional politics of the health service tribes. The challenge is to convince policy makers that a person-centred, clinically- and cost-effective resource, such as ADRe, deserves support. If policy-makers push inspection bodies, and in due course the local government commissioners (who fund much care on a means-tested basis), to demand robust medicines monitoring in care homes, as an indicator of safe or good practice, then better control of ADRs becomes realistic. What gets documented gets done. We have consistently found that less than half of potential problems were documented in patients’ notes until ADRe was introduced [29]. Only some 5% of serious ADRs are reported via voluntary systems, such as the UK’s “yellow cards” [51], and reports are vulnerable to respondent and notoriety biases [52,53]. Addressing these systemic failings will require government to show the political will to go beyond lip service to better medicines management [54,55] and take concrete steps to make it happen.

## 8. A Professional Blind Spot: Out of Sight, Out of Mind

Identifying the unintended consequences of prescribing, particularly amongst vulnerable groups, is a challenge for those regulating the marketing of medicines; however, regulators, like individual prescribers, depend on the recording of patients’ responses to medicines [56]. Large electronic databases hold information on drug utilisation, mortality, medical diagnoses and reasons for hospital admission, but outcomes important to patients, such as continence, pain and xerostomia, are rarely recorded [29].

This system-level blind spot is echoed at the level of individual practitioners: not all practitioners have memorised all the possible adverse effects of the medicines they prescribe, dispense and administer. ADRe tries to democratise that knowledge in its supporting information, ensuring carers, nurses and pharmacists have a voice in key clinical decisions concerning medicines management and alerting them to possible irregularities or even errors in prescribing. However, in the context of professional monopoly, sharing knowledge with other professions is not always welcomed [8].

Recognizing ADRs often depends on close engagement with patients over time, requiring healthcare professionals to observe and understand behaviour (e.g., posture and movement disorders, responses to pain) in ways that highlight social and cultural differences between patient and professional. Identifying a causal connection between an ADR and a behavioural sign is a complex, highly contingent social process, involving multi-faceted clinical and common-sense judgements. ADRe has a crucial role to play in addressing these challenges, creating an appropriate “index of suspicion” by equipping practitioners with the necessary supporting information.

Social and geographical distances between prescribers and patients pervade the contemporary UK healthcare system. Social, cultural and ethnic differences create barriers in communication and prescribers’ understanding of the impact of medicines on the physical and mental wellbeing of patients, sometimes leading to stereotyping and reducing the role of the patient to “other” [57,58,59]. Studies suggest that age is a factor in the way doctors interact with patients, and that older patients may be disadvantaged [60,61,62]. Such a profound and systemic challenge creates, as its logical consequence, distances between the prescribing or de-prescribing event and clinical outcomes, removing ADRs from the medical gaze. These problems increase with distance, intensifying health inequalities and resulting in ever-sharper divides in the quality of care between socially and geographically distinct populations.

## 9. ADRs and the Health Divide

UK primary care is characterised by the inverse care law [63], whereby resources are diverted away from areas of social deprivation and the greatest medical need. General practitioners (GPs) in the most deprived areas are responsible for more patients and are under greater pressures and risk of burnout [64] Socio-economic deprivation increases the use of prescription medicines and the associated risks of adverse side effects. For example, prescription of antipsychotics [65] and polypharmacy (≥10 medicines) [66] are concentrated in more deprived socio-economic groups, leading to concerns that ADRs are contributing to health inequalities [67]. The gap between rich and poor in life expectancy and healthy life expectancy is increasing, with a difference of up to 9.5 years of life expectancy between the least and most deprived areas of England [68]. The increasing impact of deprivation highlights the need for interventions that can help mitigate the social distance between doctors and certain patient groups. Accordingly, a resource like ADRe, which can be used by care assistants, is an important strategy to democratise knowledge and improve care for the most disadvantaged.

The increased demand for healthcare has been addressed by role expansion of non-medical healthcare professionals, including nurses [69]. A higher proportion of prescription items are initiated by nurses in areas of socio-economic deprivation and where the number of GPs per 100,000 population falls below 60 [70]. The limited dissemination of medical knowledge outside the body of medical professionals here makes a crucial impact; nurses, even when qualified to prescribe, do not have the same training and background knowledge of pharmacology as doctors, and substitution confined to disadvantaged areas has been a concern. Practice guidelines and systematic approaches may support practitioners and enhance care in relatively unfamiliar professional territory [71], and ADRe offers such a strategy for adults prescribed commonly-used medicines in primary care.

## 10. Conclusions

ADRs present the current UK healthcare system with stark challenges. The social imbalances between prescribers, patients, and non-prescribing healthcare professionals mean that the physical and mental wellbeing of patients is often neglected and hampers a truly multidisciplinary response to ADRs. Not all patients are served equally by this system, with medical resources more thinly spread across older and poorer sections of the population. Moreover, the knowledge that would allow non-medical healthcare professionals to meet the shortfall is not adequately disseminated, leaving nurses and other carers “out of the loop”.

ADRe offers a cost-effective step towards meaningful systemic change. It comprehensively documents the multivalent problems patients may be experiencing, giving nurses a knowledge framework that enables them to present evidence to prescribers. Beyond this, it may offer a means of addressing the iatrogenic harms and waste identified by researchers [72]. ADRe offers a real chance of bridging the divides inherent in the present healthcare system and the underlying inequalities that create them.
